# PPARs, RXRs, and Drug-Metabolizing Enzymes

**DOI:** 10.1155/2009/589626

**Published:** 2010-03-30

**Authors:** James P. Hardwick, John Y. L. Chiang

**Affiliations:** Department of Biochemistry and Molecular Pathology, Department of Integrative Medical Sciences, Northeastern Ohio Universities Colleges of Medicine and Pharmacy (NEOUCOM/NEOUCOP), 4209 State Route 44, Rootstown, OH 44272, USA

This special issue of PPAR Research is dedicated to “PPARs, RXRs and Drug Metabolizing Enzymes”. Knowledge of PPAR biology, over the past five years, has dramatically increased our understanding of the potential therapeutic usefulness of these receptors in metabolic alterations associated with disease process of alcoholic and non-alcoholic fatty liver disease and metabolic syndrome. In addition, the utility of PPAR agonist in the treatment of liver disease by normalizing hypertriglyceridemia, dyslipidemia, and the toxic effects of bile acids has a sound scientific basis in the ability of PPAR receptors to control lipid oxidation and disposal as well as regulators of hepatic inflammation. Further insight into both the indirect and direct effects of dual and pan PPAR agonist may potentiate the development of new therapeutic modalities to treat fatty acid oxidation disorders, dyslipidemia, inflammation, and bile acid accumulation associated with several liver diseases and metabolic syndrome. 

Articles included in this special issue highlight the importance of PPARs and RXR in drug metabolism and hepatic diseases associated with metabolic disorders. Alterations in drug metabolizing gene expression in different disease states due to the differential expression of PPAR isoforms highlights the importance of PPAR in disease progression and as therapeutic target in the amelioration of disease progression. The first review summarizes past and new developments in alcoholic fatty liver disease (ALD) and how PPAR/RXR regulates phase I enzymes in alcohol metabolism and the redox balance of cells. The direct impairment of PPAR by acetaldehyde results in reduction of NAD+ pool leading to alterations in lipid metabolism, increased oxidative stress, and increased pro-inflammatory cytokines, chemokines and acute phase proteins, which may be central in the onset and perpetuation of mechanism in the clinical progression of alcoholic liver disease (ALD). A second review focuses on the role of PPAR isoforms in the regulation of bile acid and cholesterol metabolism, with specific insight into how PPAR*α* regulates bile acid synthesis, conjugation, and transport by phase II and III enzymes. This review also provides an updated report on how PPARs regulate cholesterol synthesis, absorption, and reverse cholesterol transport, and how PPAR agonist may be used to treat cholestatic liver disease. This review also raises important questions concerning the use of PPAR agonist in treating bile acid accumulation in several liver diseases, which leads to hepatocyte injury, impaired liver metabolic function, progressing to liver fibrosis and cirrhosis. Further research needs to focus on how selective PPAR agonist and isoforms regulated bile acid conjugation and thereby prevent bile acid toxicity observed in cholestastic disease. The conjugation of bile acids by UDP-glucuronosyltransferase, a PPAR target gene further emphasizes the importance of PPAR in bile acid toxicity. In addition, induction of the sulfotransferase genes by PPAR and sulfonation of cholesterol in keratinocyte differentiation suggests that these phase II genes have an important role in epidermal wound healing. Finally, although numerous studies in animal models of disease have dramatically increased our understanding of how PPARs not only regulate drug metabolism and the elimination of drugs and toxic endogenous metabolities in disease progression, the application of these results to human in the design of effective PPAR agonist has all too often led to adverse drug interactions and unacceptable drug toxicity. A fourth review in this series details the exciting possibility of using a human hepatocyte chimeric mouse model to predict metabolism and possible effectiveness of new PPAR agonist in humans. The investigators clearly show the utility of this model system by the increased phosphatidylcholine transport into bile canaliculi through induction of human ABCB4 transporter by benzafibrate activation of PPAR. There are marked species differences in genes and proteins associated with the absorption, distribution, metabolism, and excretion (ADME) of xenobiotics and drugs, thus the human hepatocyte chimeric mice may not only be used for increasing the safety and effectiveness of lead drugs, but may also serve as a model system to study human liver disease and assess the potency and efficacy of dual and pan PPAR agonist in preventing or delaying disease progression. The final review in these series details how fatty acids are partitioned by fatty acid transport proteins to either anabolic or catabolic pathways regulated by PPARs, and explores how medium chain fatty acid (MCFA) *CYP4A* and long chain fatty acid (LCFA) *CYP4F* *ω*-hydroxylase genes are regulated in fatty liver. The authors propose a hypothesis that increased *CYP4A* expression with a decrease in *CYP4F* genes may promote the progression of steatosis to steatohepatitis. 

We thank the editors for the opportunity to share with other investigators these interesting reviews on drug metabolizing genes regulated by PPAR/RXR. It is apparent that drug metabolizing genes not only play a pivotal role in drug efficacy, drug toxicity, and adverse drug reactions, but also a critical and crucial role in the development and progression liver disease. Therefore, understanding how the PPAR genes and other hormone nuclear receptors are regulated during disease processes will provide us the opportunity to design effective therapeutic modalities to treat disease by the inactivation, conjugation, and transport of toxic endogenous metabolities. 

*James P. Hardwick* *James P. Hardwick* *John Y. L. Chiang* *John Y. L. Chiang*

